# Lipid Dysmetabolism in Canine Chronic Liver Disease: Relationship Between Clinical, Histological and Immunohistochemical Features

**DOI:** 10.3390/vetsci12030220

**Published:** 2025-03-02

**Authors:** Verena Habermaass, Yuki Takami, Takeshi Izawa, Francesca Abramo, Corrado Biolatti, Veronica Marchetti

**Affiliations:** 1 Department of Veterinary Sciences, University of Pisa, Via Livornese Lato Monte, 56122 Pisa, Italy; francesca.abramo@unipi.it (F.A.); veronica.marchetti@unipi.it (V.M.); 2 Laboratory of Veterinary Pathology, Osaka Metropolitan University, 1-58 Rinku-Ourai-Kita, Izumisano-shi, Osaka 598-8531, Japan; sx23628t@st.omu.ac.jp (Y.T.); takeshi.izawa@omu.ac.jp (T.I.); 3 Independent Researcher, 6340 Baar, Switzerland; corrado.biolatti@gmail.com

**Keywords:** lipid, liver, cholestasis, obesity, canine, immunohistochemistry, steatosis

## Abstract

Chronic liver diseases (CLDs) in dogs are progressive conditions that often lead to liver failure. Metabolic dysfunctions such as cholestasis, obesity, hyperlipidemia, and endocrine disorders play a key role in human liver diseases like MASLD (Metabolic Dysfunction Associated Steatotic Liver Disease) and MASH (Metabolic Dysfunction Associated Steatohepatitis), but their significance in canine CLDs is poorly understood. This study aims to evaluate the association between hepatic lipid accumulation and inflammation or fibrosis in canine CLDs and its potential association with metabolic dysfunctions. Sixteen client-owned dogs with CLDs were assessed for clinical data, histological features, and liver immunohistochemistry (IHC). Histological and IHC markers of inflammation (Iba-1, iNOS, NF-κB), fibrosis (CD206, α-SMA, Sirius Red), and lipid accumulation (adipophilin) were assessed to identify correlations with clinical conditions. The applied markers showed effectiveness in their use on canine liver tissue. Adipophilin-marked lipid accumulation correlated positively with inflammatory markers, indicating a link between steatosis and inflammation. Metabolic dysfunctions were linked to hepatic lipid accumulation and inflammation. These findings show a potential alignment of canine CLDs with human MASLD/MASH, where lipid-induced inflammation drives disease progression. IHC markers could effectively assess these processes, suggesting potential for guiding diagnostics and therapies, though further research is needed to clarify clinical associations.

## Simple Summary

Chronic liver diseases in dogs are common and progressive, often leading to liver failure, but the role of metabolic issues such as obesity, increased blood lipids, and hormonal imbalances in these conditions is not well understood. This study aimed to explore the connection between fat accumulation in the liver, inflammation, and scarring, and how these might be linked to metabolic problems. Sixteen dogs with liver disease underwent hepatic histological and immunohistochemical tissue analysis to detect signs of inflammation, fat accumulation, and scarring. The results showed that fat accumulation in the liver was linked to inflammation, suggesting a connection between liver fat and disease progression. Dogs with metabolic issues were more likely to present fat accumulation and inflammation in their livers. These findings suggest that canine liver diseases may share similarities with human conditions, where fat-induced inflammation worsens the disease. These results highlight the potential importance of metabolic conditions in canine chronic liver disease. Understanding these connections could lead to better diagnostic tools and targeted treatments for dogs, improving their health and quality of life.

## 1. Introduction

The term “Chronic Liver Disease” (CLD) in dogs refers to a group of clinical conditions that results in the progressive and possibly long-term damage to the liver, often leading to fibrosis, cirrhosis, and eventually liver failure. These conditions are characterized by persistent liver inflammation, hepatocellular damage, and circulatory and biliary disorders [1,2]. Diagnosing CLDs usually involves blood tests, diagnostic imaging, and liver histology [1,2]. In most of the CLDs, hepatocyte injury is usually represented by necrosis/apoptosis but also cellular swelling. In case of hepatocyte swelling, it is not uncommon to find glycogenic degeneration and steatosis [3,4,5,6]; however, their significance is often unknown [6]. Hepatocellular steatosis represents an accumulation of tryglicerids containing vacuoles within the cytoplasm of hepatocytes. Hepatocellular steatosis can occur because of several conditions resulting in an altered metabolism of hepatic lipids and glucose. Excess of fatty acids into the liver derived from dietary fat/carbohydrate intake or from adipose tissue mobilization of triglycerides (i.e., starvation, diabetes mellitus) can cause hepatic steatosis. Other pathological conditions that lead to a lack of energy to oxidate fatty acids (i.e., hypoxia, mitochondrial toxic damage) or to an increased fatty acid esterification to triglycerides as a consequence of hyperglycemia and hyperinsulinemia (i.e., hyperadrenocorticism) can also cause hepatic steatosis. In addition, a decreased apoprotein synthesis (dietary deficiency, hepatotoxins, and toxic drugs) or an impaired secretion of lipoproteins from the liver caused by secretory defects (hepatotoxins and toxic drugs) can cause a triglycerides accumulation in hepatocytes [6].

In humans, metabolic dysfunction-associated steatotic liver disease (MASLD), previously defined as non-alcoholic fatty liver disease (NAFLD), and metabolic dysfunction-associated steatohepatitis (MASH), previously defined as non-alcoholic steatohepatitis (NASH), are common liver diseases characterized by the accumulation of fat in the liver of individuals who have restricted alcohol consumption and in the absence of other secondary causes [7]. The mechanisms underlying MASLD and MASH are complex and multifactorial. The latest nomenclature underlines the role of metabolic syndrome in the establishment and progression of these kinds of CLDs [7]. In contrast to the well-established diagnostic criteria for MASLD/MASH in humans [8,9,10], the relationship between hepatocellular modification and metabolic alterations in canine CLDs is poorly understood.

According to veterinary medicine, canine liver steatosis is mostly considered a nonspecific sign of hepatocellular injury, which may cause hepatocyte swelling, hepatic enlargement, and intrahepatic cholestasis due to the compression of biliary canaliculi [6]. There is little information regarding the potential association of lipid dysmetabolism, hepatic inflammation, and fibrosis in dogs. However, dyslipidemia and hyperlipidemia are commonly encountered in canine patients with liver diseases, in relation to primary endocrine disorders, genetic or dietary factors, and cholestasis [11,12,13].

Thus, the study of histological and immunohistochemical (IHC) markers of inflammation, fibrosis, and lipidic accumulation in association with metabolic disorders and dyslipidemia could add important steps for the understanding of pathological mechanisms of development and progression. Our hypothesis is that the hepatocellular lipid accumulation could be associated with higher inflammatory or fibrosis processes in dogs presenting an endocrine disorder, obesity, hyperlipemic or cholestatic conditions.

Our primary aim was to immunohistochemically test markers of classically and alternatively activated macrophages, histiocytes, activated HSCs and myofibroblasts, NF-κB, and adipophilin to investigate their possible implication in liver injury, inflammation, fibrosis, and lipid accumulation in canine naturally occurring CLDs. As a second aim, we evaluated the eventual association between IHC markers and histological patterns, concurrent endocrine disorders, hyperlipemia, and obesity in CLDs dogs.

## 2. Materials and Methods

### 2.1. Animals: Enrollment and Subgrouping

**Figure 1:**
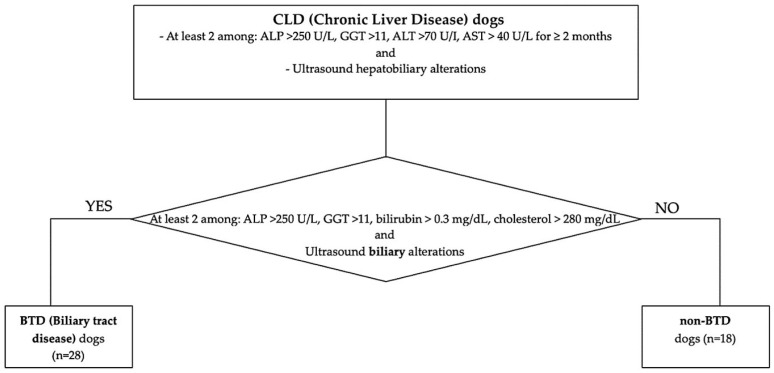
Flowchart of the study population classification, according to CLD and BTD/non-BTD.

Client-owned dogs referred to the Internal Medicine Service of the Veterinary Teaching Hospital of the University of Pisa between January 2021 and January 2023 with a diagnosis of chronic liver disease (CLD) were retrospectively included. The diagnosis of CLD was based on the concurrent presence of persistent increased liver enzymes (>2 months), specifically at least 2 among: alkaline phosphatase (ALP) > 250 U/L (reference range 45–250 U/L), gamma-glutamyl transferase (GGT) >11 (reference range 2–11 U/L), alanine aminotransferase (ALT) >70 U/I (reference range 20–70 U/L), aspartate aminotransferase (AST) > 40 U/L (reference range 15–40 U/L) and ultrasound chronic hepatobiliary alterations, such as hyperechoic parenchyma, abnormalities of hepatic dimension and/or margins, presence of nodular hepatic lesions referred to benign hyperplasia, thickened, hyperechoic and irregular gallbladder walls, abnormality of gallbladder contents (e.g., mucocele, non-gravity dependent biliary sludge, cholelithiasis), chronic intrahepatic biliary tree or common biliary duct dilatation, mineralization of the intrahepatic biliary tree. Medical reports were reviewed. A clinical exam with an evaluation of Body Condition Score (BCS 1–9 scale) and comorbidities, hematobiochemical parameters, and ultrasonographic features was registered. According to their nutritional status, dogs with BCS > 5/9 were considered “overweight”. Particular attention was pointed to the presence of hyperlipemia, identified in the case of 12h-fasting serum cholesterol > 280 mg/dL (reference range 120–280 mg/dL) and/or triglycerides > 90 mg/dL (reference range 25–90 mg/dL), eventual underlying endocrinopathies (hyperadrenocorticism, hypothyroidism, diabetes mellitus), or evidence of biliary tract involvement. Biliary tract disease (BTD) was identified in the case of two or more of the following laboratory alterations: ALP > 250 U/L (reference range 45–250 U/L), GGT > 11 U/L (reference range 2–11 U/L), total bilirubin > 0.3 mg/dL (reference range 0.07–0.3 mg/dL), cholesterol > 280 mg/dL (reference range 120–280 mg/dL), and one or more concurrent ultrasonographic biliary tract alterations [14]. Ultrasonographic BTD features considered were identified in the case of thickened, hyperechoic, and irregular gallbladder walls; abnormality of gallbladder contents (e.g., mucocele, non-gravity-dependent biliary sludge, cholelithiasis); chronic intrahepatic biliary tree or common biliary duct dilatation; and mineralization of the intrahepatic biliary tree. According to this classification, enrolled CLD dogs were divided into subgroups according to biliary tract involvement, specifically BTD and non-BTD (Figure 1). Liver histology features and IHC results between dogs with and without hyperlipemia, endocrine disorders, BTD, or increased BCS were compared.

### 2.2. Histology and Immunohistochemistry

All dogs were submitted to liver biopsy and histopathological examination as a part of the individual diagnostic work-up or in the context of an elective or urgent/emergency cholecystectomy. Laparoscopic and/or laparotomic (punch 5 mm technique) liver biopsy samples were fixed in 10% neutral buffered formalin, processed routinely, and embedded in paraffin wax. Liver biopsies (4–6 biopsies for each case) were stored as formalin-fixed paraffin-embedded (FFPE) blocks and subsequently analyzed by expert veterinary pathologists from the University of Pisa (Italy) (F.A.) and the Osaka Metropolitan University of Osaka (Japan) (T.I.; Y.T.). Histological and immunohistochemical scoring was performed by two board-certified veterinary pathologists (YT and TI; Diplomates Japanese College of Veterinary Pathologists).

FFPE (Formalin-Fixed Paraffin-Embedded) blocks were sectioned at 5 µm in thickness. The deparaffinized serial sections of the liver were stained with hematoxylin and eosin for histopathological examination and for IHC analyses. The histopathological diagnosis was made on the basis of the guidelines for the interpretation of liver biopsies and the most recent scientific literature. For IHC, after dewaxing and pretreatment, tissue sections were stained by the Histostainer (Nichirei Biosciences, Tokyo, Japan). Sections were incubated with 5% skimmed milk for 15 min, followed by 1 h incubation with primary antibodies as listed in Table 1.

After treatment with 3% H_2_O_2_ for 15 min, anti-mouse or anti-rabbit secondary antibody (Histofine Simple Stain MAX PO^®^; Nichirei Biosciences) was applied for 1 h. Visualization was made with 3,3′-diaminobenzidine (DAB, Nichirei Biosciences DAB substrate kit). Sections were counterstained lightly with hematoxylin. Perls’ Prussian blue counterstain was performed for selected slides in order to differentiate between IHC signals and iron accumulation, as DAB and iron granules are similar in color in the sections with conventional IHC. FFPE liver tissue specimens were immunostained for multiple histiocyte markers, which are differently associated with the development of pathological mechanisms. In more detail, IHC for iNOS (classically activated or M1 macrophages), CD206 (alternatively activated or M2 macrophages that stimulate collagen deposition and indirect markers of fibrosis), Iba-1(pan-macrophage marker), and NF-κB (pro-inflammatory marker) were used. Liver fibrosis was investigated by Sirius red staining (for collagens) and IHC for αSMA (activated HSCs and myofibroblasts). Adipophilin antibody was used to assess the amount of intracellular lipid accumulation.

Histological hematoxylin-eosin (HE) staining was used to assess inflammation levels. Each dog was assigned a semi-qualitative score ranging from 0 to 3 to indicate the degree of inflammation focused on the hepatic area affected: a lobular-parenchymal (LP) score reflecting the intensity of inflammation in the lobule area, a portal-peribiliary (PP) score, and a general (G) HE inflammation grading given by the sum of LP and PP inflammation areas. The histological presence of hepatic stellate cells (or HCS) vacuolation and ductular reaction were also evaluated through HE and similarly scored as 0–3 and present/absent, respectively. The scoring was performed by two board-certified veterinary pathologists (YT and TI; diplomates of the Japanese College of Veterinary Pathologists).

Similarly, to assess responses for various immunohistochemistry (IHC) antibodies, including CD206, iNOS, α-SMA, Iba-1, NF-κB, and Adipophilin, as well as for Sirius Red staining, each dog was assigned a semi-qualitative score ranging from 0 to 3 to indicate the degree of reaction intensity, with 0 representing an absence of reaction, 1 for mild reactions, 2 for moderate reactions, and 3 for severe reactions. These scores were similarly and differently assessed considering the positivity in LP and PP areas, with a general (G) grading given by the sum of LP and PP scores. Composite inflammation scores for each region were created by summing the individual scores for Iba-1, iNOS, and NF-κB, respectively, to generate G, LP, and PP IHC inflammation scores. IHC Fibrosis scores were derived using a similar summation approach, combining scores of CD206, α-SMA, and Sirius Red for the G, LP, and PP regions, respectively. Adipophilin staining was separately evaluated as a specific marker of lipid accumulation within hepatocytes [18,19], without a scoring differentiation according to the hepatic area affected.

### 2.3. Statistical Analysis

Statistical analyses were conducted using R software (version 4.4.2). To explore correlations among the single immunohistochemistry (IHC) markers and Sirius Red staining, Pearson correlation coefficients were computed. Additionally, a principal component analysis (PCA) was performed based on the covariance matrix of these markers to capture patterns of variability. Further analysis was carried out by evaluating IHC marker correlations within the HE and IHC inflammatory/fibrosis score in the G, LP, and PP liver regions. For each region, composite inflammation scores were generated by summing the IHC scores of Iba-1, iNOS, and NF-κB for LP inflammation and following the same approach for PP inflammation. Similarly, fibrosis scores were created by combining the scores of CD206, α-SMA, and Sirius Red for both LP and PP regions. In addition, histological (HE) assessments were used to evaluate inflammation in the LP and PP regions, yielding LP HE and PP HE inflammation scores, respectively. The relationships between IHC and HE inflammation and IHC fibrosis scores, as well as their correlations with other variables (such as the presence or absence of cholestasis, hyperlipemia, endocrine disorders, obesity, ductular reaction, and stellate cells), were further examined. To this end, Pearson correlation coefficients were calculated to assess the relationships between continuous scores. For the binary variables, point-biserial correlation coefficients were applied.

## 3. Results

### 3.1. Animals

A group of sixteen dogs with different kinds of CLDs was included. The majority were mix-breed (n = 8; 50%), followed by toy poodle (n = 2; 13%), and one of each (6%) of the following breeds: West Highland White Terrier, Zwergpincher, Beagle, Border Collie, Dachshund, and Golden Retriever. The median age was 9.5 (1.8–15); 11/16 (69%) were female (5/11 neutered), and the remaining 5 (31%) were intact males.

Five dogs out of 16 (31%) were diagnosed with an endocrinopathy (diabetes mellitus, n = 3; hyperadrenocorticism, n = 1; hypothyroidism, n = 1); 10/16 (62.5%) dogs presented hyperlipemia, 8/16 (50%) dogs presented BTD according to the applied criteria, and 9/16 (56%) presented increased BCS. Signalment, clinical and biochemical findings, ultrasound hepatobiliary, and histological diagnosis are reported in Table 2 and Table 3.

### 3.2. Histological and Immunohistochemical Features

**Figure 2:**
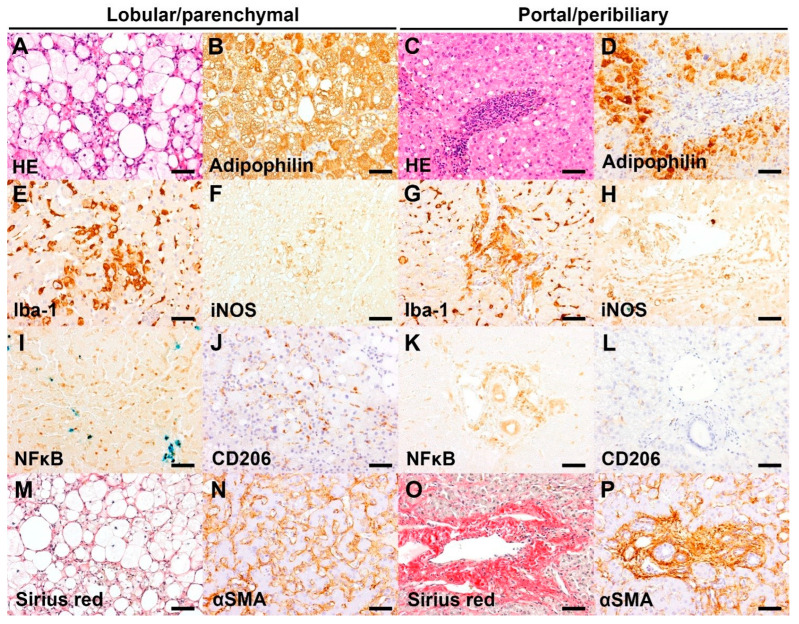
Representative images of hepatic histopathology and immunohistochemistry (IHC). Bar = 50 μm. (**A**) Infiltrate of foamy macrophages and a few neutrophils in the hepatic parenchyma, associated with marked microvesicular and macrovesicular steatosis of hepatocytes. HE, case 1 (Inflammation score 3). (**B**) The microvesicular and macrovesicular steatosis in the centrilobular hepatocytes are delineated by adipophilin IHC. IHC for adipophilin, counterstained with hematoxylin, case 1 (IHC score 3). (**C**) Marked neutrophil infiltrate admixed with lymphoplasmacytic infiltrate in the portal area. HE, case 10 (Inflammation score 3). (**D**) Absence of adipophilin immunolabeling in the portal area with an accumulation of adipophilin-positive lipid vacuoles in the periportal hepatocytes. IHC for adipophilin, counterstained with hematoxylin, case 10 (IHC score 1). (**E**) Increased number and hypertrophy of Iba-1 positive Kupffer cells in the sinusoids. IHC for Iba-1, counterstained with hematoxylin, case 14 (IHC score 3). (**F**) Aggregates of iNOS-positive macrophages/Kupffer cells in the hepatic parenchyma. IHC for iNOS, counterstained with Prussian blue, case 14 (IHC score 2). (**G**) Marked infiltrates of Iba-1 positive macrophages in the portal area. IHC for Iba-1, counterstained with hematoxylin, case 12 (IHC score 3). (**H**) Presence of iNOS-positive macrophages in the portal area. IHC for iNOS, counterstained with Prussian blue, case 14 (IHC score 2). (**I**) Infiltrate of inflammatory cells (morphologically lymphocytes or Kupffer cells) with nuclear NFκB immunolabeling in the sinusoids. Aggregates of iron-laden Kupffer cells/macrophages are also present. IHC for NFκB, counterstained with Prussian blue, case 16 (IHC score 3). (**J**) Infiltrate of CD206-positive Kupffer cells in the sinusoids. IHC for CD206, counterstained with hematoxylin, case 1 (IHC score 2). (**K**) Infiltrate of inflammatory cells (morphologically macrophages) with cytoplasmic NFκB immunolabeling in the portal area. IHC for NFκB, counterstained with Prussian blue, case 12 (IHC score 2). (**L**) Absence of CD206-positive cells in the portal area. IHC for CD206, case 4 (IHC score 0). (**M**) Presence of Sirius red-positive collagen fibers surrounding the swollen hepatocytes with lipid vacuoles. Sirius red stain, case 14 (Score 1). (**N**) αSMA-positive activated hepatic stellate cells along the sinusoids. IHC for αSMA, counterstained with hematoxylin, case 12 (IHC score 2). (**O**) Portal fibrosis extending into the adjacent tissue. Sirius red, case 3 (Score 2). (**P**) A number of αSMA-positive activated myofibroblasts along the portal fibrosis. IHC for αSMA, counterstained with hematoxylin, case 3 (IHC score 3).

Histological HE findings (ductular reaction, HSC vacuolation) and HE inflammation grades according to LP and PP areas are reported in Table 4. Representative images of hepatic HE and IHC of selected cases are shown in Figure 2. Similarly, Table 5 presents the individual grades (0–3) assigned to CLD dogs based on the positivity of various IHC markers (Iba-1, iNOS, NFκB, α-SMA, CD206) and Sirius Red staining. These grades reflect the specific positivity within the lobular-parenchymal (LP) and portal-peribiliary (PP) areas and the overall positivity in general hepatic tissue (G), given by the sum of LP and PP positivities. Additionally, Table 5 includes the grades (0–3) for hepatic tissue adipophilin positivity for each dog.

Composite IHC grades of inflammation and fibrosis for each dog, considering hepatic areas (G, LP, and PP), are reported in Table 6.

The present cases with CLD had a various degree of LP and/or PP inflammation. Moderate to severe PP inflammation was present in 7/16 dogs, which is associated with the presence of ductular reaction (oval cell hyperplasia). The PP inflammation is characterized by diffuse neutrophil infiltrate in the portal area and/or around the bile duct, admixed with lymphoplasmacytic infiltrate (Figure 2C). Iba-1 positive macrophages were often infiltrating into the PP lesions (Figure 2G). Moderate to severe LP inflammation was present in 4/16 dogs and was characterized by infiltrate of hypertrophied (activated) Kupffer cells in the sinusoids (Figure 2E), often with formation of microgranuloma consisting of hemosiderin-laden macrophages. Moderate immunoreactivity for iNOS was observed in the Kupffer cells and/or portal macrophages (Figure 2F,H) of 6/16 dogs. CD206 immunoreactivity was weak or absent in the sinusoids and absent in the portal area (Figure 2J,L). Moderate to severe NFκB immunoreactivity was present in the LP and/or PP lesions of 13/16 dogs (Figure 2I,K). Moderate PP fibrosis was present in 14/16 dogs (Figure 2O), which was associated with proliferation and infiltration of myofibroblasts in the PP area (Figure 2P). Fibrosis in the LP area was absent or very weak (Figure 2M), and a few cases (3/16) had a moderate immunoreactivity for α-SMA in the sinusoids (Figure 2N). Moderate to severe hepatocyte steatosis, visualized by adipophilin immunohistochemistry, was present in 4/16 dogs (Figure 2A,B,D). Vacuolation of hepatic stellate cells was present in 3/16 dogs.

**Figure 3:**
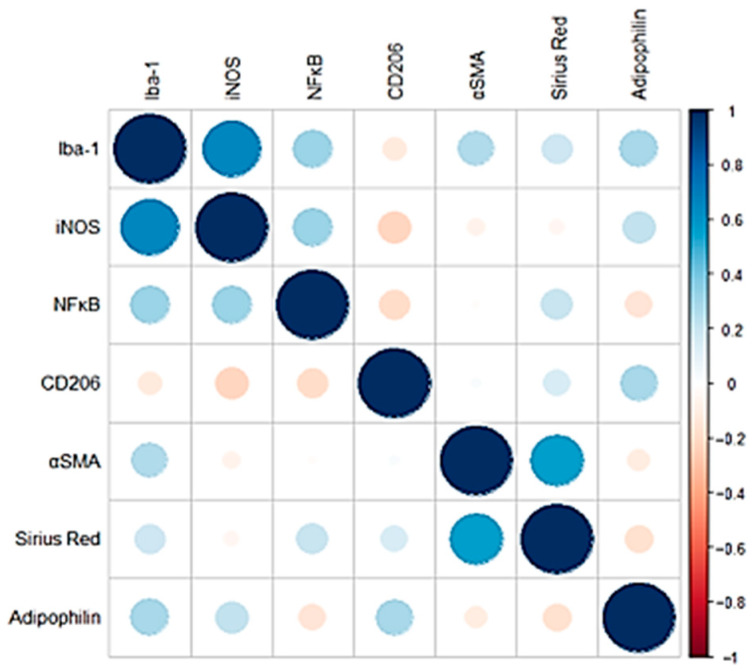
Pearson correlation coefficients of the IHC and histological markers analyzed. Negative correlations are shown in red, while positive correlations are shown in blue. The size of each circle is proportional to the magnitude of the correlation.

Correlation analysis of the IHC inflammation markers (Iba-1, iNOS, NF-κB) revealed that all markers exhibited a moderate positive correlation with each other. Positive correlations were also observed between fibrosis markers (α-SMA, CD206, Sirius Red), although in this case the magnitude of the correlation appeared to be less pronounced (Figure 3). Adipophilin showed mild-moderate positive correlations with inflammatory markers such as iNOS and Iba1 and with CD206 (fibrosis marker).

**Figure 4:**
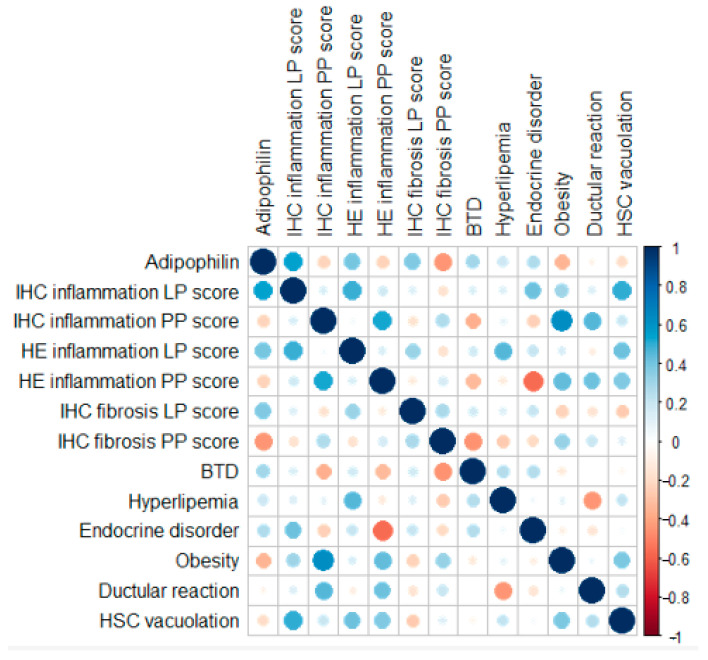
Pearson correlation coefficients and point-biserial correlation coefficients of the IHC and binary markers analyzed. Negative correlations are shown in red, while positive correlations are shown in blue. The size of each circle is proportional to the magnitude of the correlation.

A spatial dependency for inflammation was evident (Figure 4). LP IHC inflammation scores showed a moderate correlation with LP HE inflammation scores but not with PP IHC inflammation scores. Conversely, PP IHC inflammation scores correlated moderately with PP HE inflammation scores. Fibrosis scores, however, did not demonstrate major correlations with any of the tested variables. PP inflammation scores, assessed through both IHC and HE methods, were moderately positively correlated with the presence of obesity and ductular reaction. The presence of lobular-parenchymal, both IHC and HE-detected inflammation, was moderately positively correlated with the histological evidence of stellate cell vacuolation.

## 4. Discussion

According to our results, IHC for Iba-1, iNOS, NF-κB, CD206, and αSMA showed to be effective in their application on canine liver tissue. Interestingly, our hypothesis of an association between hepatic lipid accumulation and inflammation seems to be confirmed based on these preliminary results,

Iba-1 is a marker primarily used to identify microglia in the central nervous system [20], but its application has extended to studying histiocytes in various tissues, including the liver. In chronic liver disease, Iba-1 IHC is employed to investigate the role of macrophages in inflammation and fibrosis [21]. Iba-1 is currently used in mouse models of MASLD/MASH [21,22], however, in dogs, it is mainly applied in canine oncologic disorders such as hystiocitic neoplasia [23] to evaluate the expression of different macrophage subtypes. iNOS is an enzyme that produces nitric oxide (NO) under inflammatory conditions. In chronic liver disease, persistent inflammation leads to the upregulation of iNOS expression, contributing to liver damage, fibrosis, and disease progression. Application of iNOS IHC is a valuable tool in studying human chronic liver diseases, such as cirrhosis and chronic hepatitis [24]. In dogs, iNOS activity was mostly evaluated in association with septic shock, systemic inflammatory, and infective responses [25,26,27]. More recently, it was shown to be an effective marker of liver inflammation in high-fat-fed obese dogs [28]. In natural-occurring canine hepatic disorders, a higher positivity for hepatic iNOS was reported in dogs with liver disease compared to healthy controls [29] and its positive correlation with the grade of necroinflammatory activity [29]. NF-κB IHC is a valuable tool in studying liver diseases, particularly in understanding the role of inflammation and immune responses in liver pathology. NF-κB is a transcription factor that regulates genes involved in immune and inflammatory responses. Studies show that NF-κB is often activated in hepatocytes and Kupffer cells in hepatitis, contributing to the inflammatory response and disease progression [30]. In liver fibrosis, NF-κB IHC helps in identifying its role in the activation of HSCs, which are central to the development of fibrosis. In dogs, an overexpression of NF-κB was demonstrated during neoplastic diseases, similarly to humans [31]. Recently, NF-κB was evaluated in the liver of dogs with experimentally induced type 1 diabetes mellitus, showing a reduction in its activity and in serum liver enzymes in dogs treated with N-acetyl-cysteine and insulin compared to untreated dogs [32]. Literature regarding its application in dogs with naturally occurring and non-neoplastic liver diseases is scarce. CD206, also known as the mannose receptor, is a marker commonly used in IHC to identify alternatively activated (M2) macrophages. In the context of human chronic liver disease, CD206 IHC is particularly useful for studying liver fibrosis, cirrhosis, and the role of macrophages in the progression of these conditions. M2 macrophages are known to contribute to tissue repair and fibrosis by producing anti-inflammatory cytokines and promoting collagen deposition. Therefore, assessing the presence and density of CD206-positive macrophages in liver tissue can provide insights into the extent of fibrosis and the inflammatory environment within the liver [33,34]. CD206 in dogs was mainly applied to neoplastic diseases [35,36], not affecting the liver. αSMA IHC is widely used in the study of chronic liver diseases, particularly in the context of liver fibrosis. αSMA is a marker of activated HSCs, which play a central role in the fibrogenesis process by producing extracellular matrix components that lead to fibrosis of the liver. As previously reported in studies conducted in canine CLDs [37,38], we find a positivity for αSMA in hepatic areas histologically showing fibrosis.

The presence of a positive correlation between Iba-1, iNOS, CD206, and adipophilin may show how the presence of a hepatic lipidic accumulation, pointed out by the positivity for adipophilin IHC, may correlate especially with the presence of inflammatory processes. The result showing a positive correlation between immunohistochemical positivity for adipophilin and inflammatory markers (such as Iba-1, iNOS) in CLDs dogs suggests that lipid accumulation in hepatocytes is associated with higher inflammation. This correlation can be understood through insights from human medicine, where similar mechanisms are observed, particularly in conditions like MASLD and MASH. In these conditions, lipid accumulation within hepatocytes can lead to lipotoxicity. This condition arises when excess fatty acids and their metabolites induce oxidative stress, mitochondrial dysfunction, and endoplasmic reticulum stress, triggering inflammatory signaling pathways [39]. Lipid droplets marked by adipophilin may reflect this lipotoxic environment, which subsequently activates inflammatory pathways, as indicated by the positive correlation with Iba-1 and iNOS. In human MASH, activated Kupffer cells in the liver play a crucial role in promoting inflammation. Iba-1 positivity indicates macrophage activation, which can be driven by lipid-induced stress signals [21,22]. The positive correlation between adipophilin and Iba-1 suggests that lipid-laden hepatocytes may promote the recruitment and activation of macrophages, further amplifying the inflammatory response. iNOS is a key regulator of inflammation and is known to be activated by various stimuli, including lipids and reactive oxygen species. In conditions where there is excessive lipid accumulation (as indicated by adipophilin), iNOS activation can occur, leading to the production of pro-inflammatory cytokines and further perpetuating liver inflammation. In humans, the transition from MASLD to MASH is marked by the activation of inflammatory pathways, including those involving iNOS [24,30], in response to lipotoxicity. The positive correlation between adipophilin and inflammatory markers in dogs mirrors this human disease process, where lipid accumulation sets the stage for inflammatory responses that contribute to disease progression [40,41]. Further studies are needed to better understand how adipophilin may be used in dogs for assessing disease progression in chronic liver disease. Sirius Red does not seem to positively correlate with the other IHC markers used; otherwise, the correlation was mildly negative. In early-stage steatosis, lipid accumulation is prominent, whereas fibrosis might develop later or under different pathological stimuli. Human studies suggest that while initial steatosis does not directly lead to fibrosis, the progression to steatohepatitis, where inflammation and hepatocyte injury are present, is more likely to trigger fibrosis [40]. The negative correlation could therefore represent a separation of these stages, with adipophilin marking the lipid-rich but less fibrotic phase of the disease and Sirius Red staining marking the fibrotic phase where lipid content has diminished.

Lobular-parenchymal IHC inflammation scores showed a moderate correlation with LP HE inflammation scores but not with PP IHC inflammation scores. Conversely, PP IHC inflammation scores correlated moderately with PP HE inflammation scores, suggesting some alignment between IHC and HE assessments in detecting localized inflammation. The absence of a positive correlation between the parenchymal-lobular inflammation (both HE and IHC detected) and the portal-perilobular inflammation (both HE and IHC detected) in dogs with chronic liver disease indeed suggests that different pathogenic mechanisms could be at play in each of these liver regions. We should consider that the portal-peribiliary region (around the bile ducts and portal veins) and the lobular-parenchymal area (the primary hepatocyte-rich areas of the liver lobules) have different vascular supplies and immune cell distributions and thus, potentially different inflammatory triggers and responses to injury [42].

The absence of a spatial hepatic IHC inflammation correlation likely reflects these separate microenvironments and mechanisms of inflammation in the portal-peribiliary and lobular-parenchymal areas. This compartmentalization could indeed be a hallmark of chronic liver disease in dogs, suggesting that inflammation in these regions does not follow a single, unified pathway but rather involves different triggers, cell types, and pathogenic responses [42]. Further studies are warranted to elucidate the underlying pathogenic mechanisms, including investigations into the gut microbiota composition and microbial metabolites, such as lipopolysaccharides (LPS) and other microbe-derived molecules, which are filtered by the liver and may contribute to hepatic inflammation and immune responses [43]. Understanding these differences might provide insights into targeted treatments that address the specific types of inflammation in each region.

Portal-peribiliary inflammation scores, assessed through both IHC and HE methods, were moderately positively correlated with the presence of ductular reaction. Ductular reaction (bile duct hyperplasia) consists of the formation of new, irregular, and tortuous ductules or chains of cells formed from flattened or cuboidal basophilic epithelium [44]. The correlation between PP inflammation and ductular reaction points out how the chronic inflammation may possibly lead to ductular alteration and remodeling [45], as a pathological mechanism already recognized in dogs [46].

The presence of inflammation, both IHC and HE detected, and particularly LP IHC inflammation, was moderately positively correlated with the histological evidence of stellate cell vacuolation. The pathophysiological meaning of vacuolation of hepatic stellate is still uncertain [46]; however, HSCs usually signal metabolic changes, cellular injury, or activation in response to liver damage [47]. In our population, the presence of inflammation may have influenced the physiological metabolism of HCSs, which are also known to play an important role in the establishment and progression of the fibrotic process [38,47].

A mild to moderate positive correlation was noted between immunohistochemical (IHC) and HE LP inflammation, adipophilin positivity, and the clinical variables examined, including the presence of endocrine disorders, obesity, biliary tract disease (BTD), and hyperlipidemia. In humans, the attention on the role of lipidic and glucidic metabolisms during non-alcoholic liver diseases is constantly increasing. MASH, which represents a more severe form of MASLD, is characterized by liver inflammation and damage in addition to fat accumulation. Key risk factors include obesity, type 2 diabetes, metabolic syndrome, hyperlipidemia, and sedentary lifestyle [48]. 

Therefore, in our population of dogs with chronic liver disease (CLD), it is reasonable to hypothesize that these clinical conditions (BTD, endocrine disorders, obesity, and hyperlipidemia) may have contributed to lipid accumulation, as indicated by the positive correlation with adipophilin and to a hepatic inflammatory status. Moreover, the stronger correlation of these potential clinical risk factors with the lipid accumulation and inflammation of the LP area is consistent with what occurs in humans, where the hepatic lobular-parenchimal region is primarily affected, more so than the portal-peribiliary area. This region is highly susceptible to metabolic stress due to the relative hypoxia and is heavily involved in lipid metabolism and detoxification processes. As a result, zone 3 hepatocytes are more vulnerable to lipotoxicity and oxidative stress, which drive the progression of steatosis, inflammation, and fibrosis in MASLD/MASH [49].

Considering obesity, finding a correlation between obesity and portal-peribiliary inflammation rather than the more typical lobular-parenchymal inflammation can indeed be surprising, as MASLD/MASH often involves the lobular region first. However, there are several possible explanations. For instance, in humans, obesity is associated with chronic low-grade systemic inflammation, often marked by elevated cytokines like TNF-α, IL-6, and IL-1β. These cytokines are delivered to the liver predominantly through the portal circulation, leading to increased immune cell infiltration and inflammation in the portal-peribiliary areas [50]. This process could precede or occur independently of lobular-parenchymal injury.

This study should be considered in view of its limitations. First of all, the retrospective nature of the study and the restricted number of cases possibly did not allow for the observation of significant differences in subgroups of dogs according to clinic-pathological variables. This study represents a preliminary study aimed to evaluate the possible applicability of IHC markers in canine non-neoplastic CLDs and certainly requires future investigations. By highlighting emerging patterns, this analysis provides insights to guide future research, laying the groundwork for larger confirmatory studies that can further investigate these preliminary trends. These results need further investigations to better assess the association between lipidic metabolism, biliary tract involvement, and liver inflammatory, oxidative, and fibrotic processes.

## 5. Conclusions

The positive correlation between adipophilin and inflammatory markers such as Iba-1 and iNOS in CLDs dogs suggests that lipid accumulation within hepatocytes could be associated with an inflammatory response. Lobular-parenchimal inflammation also correlated to the presence of some clinical conditions such as obesity, biliary tract disease, endocrine disorders, and hyperlipemia. This relationship aligns with findings in human liver diseases such as MASLD and MASH, where lipotoxicity leads to the activation of inflammatory pathways. Understanding this correlation could provide insights into the pathogenesis of liver disease in dogs and potentially guide therapeutic strategies aimed at reducing both lipid accumulation and inflammation. Based on these results, IHC may be an effective tool that may provide useful insights in its application to routinary hepatic histological assessment of canine CLDs. IHC for Iba-1, iNOS, NF-κB, CD206, and αSMA showed to be effective in their application on canine liver tissue; further studies are needed to better understand their possible association with potential clinical risk factors CLDs-related.

## Figures and Tables

**Table 1:** The antibodies used for immunohistochemistry.

Antibody	Significance	Host/Clone	Dilution	Pretreatment	CatalogID	Source
CD206	Alternatively activated (M2) or reparative macrophage (collagen deposition)	Rabbit/polyclonal	1:1000	AC/Citrate	Ab64693	Abcam, Cambridge, MA, USA
iNOS	Classically activated (M1) or proinflammatory macrophage	Rabbit/polyclonal	1:500	AC/Citrate	AHP303	Bio-Rad, Hercules, CA, USA
αSMA	Activated myofibroblast/ activated HSC	Mouse/1A4	1:500	No pretreatment	M0851	Dako, Carpinteria, CA, USA
Iba-1	Kupffer cell/macrophage (pan-macrophage marker)	Rabbit/polyclonal	1:1000	AC/Citrate	019-19741	FUJIFILM Wako Pure Chemical Corporation, Osaka, Japan
NF-κB (p65/RelA)	Transcription factor for pro-inflammatory factors (cytokines, chemokines)	Rabbit /1D8	1:500	AC/Citrate	ZRB1498	Sigma-Aldrich, St Louis, MO, USA
Adipophilin (perilipin 2)	Structural component of lipid droplets, localized in their outer surface	Mouse/AP125	1:200	AC/Citrate	690102S	Progen, Heidelberg, Germany

Legenda: CD206: macrophage mannose Receptor; iNOS: inducible nitric oxide synthase; αSMA: alpha smooth muscle actin; Iba-1: ionized calcium binding adaptor molecule 1; NF-κB: Nuclear factor kappa-light-chain-enhancer of activated B cells; HSC: hepatic stellate cell [15,16,17].

**Table 2:** Clinical and biochemical aspects of the dogs enrolled; presence of biliary tract disease (BTD) and hyperlpemia, according to the applied criteria.

N	Breed	Sex	Age	Endocrinopathy	BCS	Cholesterol (150–280 mg/dL)	Triglicerids (25–90 mg/dL)	Hyperlipemia	BTD
1	Border Collie	F	7	diabetes mellitus	3	297.5	370	yes	yes
2	Dachshund	M	8.1	diabetes mellitus	4	459.1	65	yes	yes
3	Golden Retriever	F	1.8	/	5	127.2	63	no	no
4	WHWT	F	10	diabetes mellitus	5	248.4	48	no	no
5	Mix-breed	FN	10	/	5	270.6	143	yes	no
6	Mix-Breed	M	2.7	/	5	307.1	56	yes	yes
7	Zwergpinscher	M	10.4	/	6	250.5	59	no	no
8	Mix-Breed	FS	9.9	hypothyroidism	6	266.7	69	no	no
9	Mix-breed	FN	9.9	/	6	353.5	58	yes	no
10	Toy Poodle	M	6.4	/	6	250	172	yes	no
11	Mix-breed	F	9.7	/	6	167.9	83	no	yes
12	Mix-breed	FN	11.4	/	6	345.2	77	yes	yes
13	Mix-breed	FN	7.02	/	7	338	187	yes	no
14	Mix-Breed	M	9.3	/	7	808.8	717	yes	yes
15	Beagle	F	8.5	/	4	251	80	no	yes
16	Toy Poodle	F	14.8	hyperadrenocorticism	6	354	66	yes	yes

Age (years); sexual status (F: female, FN: female neutered, M: male); Body Condition Score (BCS) on 1–9 scale; serum cholesterol and triglycerides and respective ranges.

**Table 3:** Ultrasound hepatobiliary features and histological diagnosis of the dogs enrolled.

N	US Hepatobiliary Findings	Histological Features
1	Moderate diffuse liver disease with increased liver size and diffuse increase in echogenicity. Mild cholecystopathy with slightly thickened and hyperechoic walls. Common bile duct moderately dilated along its entire course (4 mm), filled with corpuscular echogenic contents, and with slightly thickened walls. Gallbladder with biliary sludge slightly corpuscular contents in suspension.	Degenerative hepatopathy (steatosis), multifocal steatohepatitis
2	Mild/moderate diffuse liver disease with slightly increased in size, increased echogenicity. Mild/moderate cholecystopathy with slightly enlarged walls thick and hyperechoic, irregular mucosal profile with the presence of a cystic mucosal lesion.	Degenerative hepatopathy (glicogenosis)
3	Moderate/severe diffuse liver disease with slightly reduced in size with irregular shape and slightly lumpy profile, heterogeneous echogenicity and altered echo structure due to the presence of multiple hyperechoic bands and striae delimiting multiple hypoechoic nodular lesions of variable dimensions deforming the capsule. Peritoneal effusion. Aberrant venous vessels compatible with shunts acquired.	Lobular dissecting hepatitis
4	Moderate diffuse liver disease with increased in size with rounded margins, diffusely increased echogenicity. Mild cholecystopathy. Gallbladder with biliary sludge and aggregated hyperechoic content suspended.	Chronic hepatitis, degenerative hepatopathy (glycogenosis)
5	Moderate diffuse liver disease with moderately increased in size, with rounded margins, moderately increased echogenicity in diffuse manner and slightly heterogeneous echo structure with the presence of multiple nodular lesions. Mineralizations of the bile/parenchymal ducts. Mild diffuse cholecystopathy. Hyperechoic structure in the lumen of the gallbladder (cholelitis, aggregated bile sludge, mucosal proliferation).	Cholangiohepatitis, intrahepatic cholestasis
6	Moderate diffuse liver disease with moderately enlarged and hyperechoic, finely dissimilar echo structure. Severe diffuse cholecystopathy with gallbladder mucocele stage VI, suspected parietal rupture.	Colangitis, portal inflammation, degenerative hepatopathy (glycogenosis), pigmentary granulomas
7	Moderate diffuse liver disease with liver slightly increased in size, slightly increased echogenicity and finely inhomogeneous echo structure with multiple lesions. Gallbladder with slight hyperechoic content in suspension	Degenerative hepatopathy (glycogenosis), portal inflammation, pigmentary granulomas
8	Moderate diffuse liver disease with liver moderately enlarged in size, with increased parenchymal echogenicity and inhomogeneous echo structure (stiae, multiple lesions). Gallbladder with biliary sludge, deposited.	Chronic hepatitis
9	Moderate diffuse liver disease, with liver with generally hyperechoic parenchyma. Mild interlobar hepatic effusion.	Portal inflammation, Intrahepatic cholestasis, degenerative hepatopathy (glycogenosis)
10	Moderate cholecistopathy, with gallbladder with abundant hyperechoic corpuscular content, partially organized, thickened and hypoechoic walls.	Chronic hepatitis, degenerative hepatopathy (glycogenosis), pigmentary granulomas
11	Mild diffuse liver disease with moderately increased in size with slight increase in echogenicity. Severe diffuse cholecystopathy with gallbladder mucocele stage VI, suspected parietal rupture. Moderate/severe multifocal peritoneal reactivity.	Cholangiohepatitis, portal inflammation, intrahepatic cholestasis
12	Moderate cholecystopathy with intraluminal cholelitis and suboccluding/occluding the common bile duct.	Colangitis with concentric periductural fibrosis, intra and extra-hepatic cholestasis, pigmentary granulomas
13	Mild diffuse liver disease, slightly increased in size.	Portal inflammation, intrahepatic cholestasis
14	Moderate diffuse liver disease, with liver slightly increased in size and diffusely hyperechoic. Severe diffuse cholecystopathy with gallbladder mucocele stage VI, suspected parietal rupture.	Chronic hepatitis, concentric periductular fibrosis, degenerative hepatopathy (steatosis)
15	Moderate diffuse liver disease, with liver with slightly hypoechoic parenchyma alteration of the echo structure for nodular lesions. Gallbladder with gravity-dependent biliary sludge and gallstones.	Portal inflammation, hepatocellular cholestasis, pigmentary granulomas
16	Moderate cholecystopathy with thickened walls and hyperechoic, presence of mild amount of peripheral anechoic fluid. Slightly dilated common bile duct and presence of a small layer of liquid near the duodenal papilla.	Cholangiohepatitis, intrahepatic cholestasis, pigmentary granulomas

**Table 4:** Histological features and individual HE grades of inflammation, considering hepatic areas.

N.	Inflammation HE PL (0–3)	Inflammation HE PP (0–3)	SUM Inflammation HE (0–6)	Ductular Reaction (A/P)	HSC Vacuolation (0–3)
1	3	0	3	A	0
2	0	0	0	A	0
3	0	2	2	P	0
4	1	0	1	P	0
5	1	1	2	P	0
6	2	2	4	P	0
7	1	2	3	P	1
8	1	2	3	P	0
9	1	1	2	A	0
10	2	3	5	P	3
11	1	2	3	P	0
12	1	2	3	P	0
13	1	1	2	A	0
14	1	0	1	P	0
15	0	0	0	P	0
16	2	1	3	P	3

Legenda: LP: lobular-parenchimal; PP: portal-peribiliary; HE: Hematoxilin & eosin; HSC: hepatic stellate cells; A: absent; P: present; Ductular reaction is classified as absent/present. SUM Inflammation HE = Inflammation HE PL + Inflammation HE PP.

**Table 5:** Individual IHC and Sirius Red grades assigned to CLD dogs, according to the general hepatic tissue positivity (G), lobular-parenchimal or portal-peribiliary areas positivity.

N.	Iba-1 (0–3)			iNOS (0–3)			NFκB (0–3)			αSMA (0–3)			CD206 (0–3)			SiriusRed (0–3)			Adipophilin (0–3)
	LP	PP	G	LP	PP	G	LP	PP	G	LP	PP	G	LP	PP	G	LP	PP	G	G
1	3	0	3	1	0	1	2	0	2	2	2	4	2	0	2	1	2	3	2
2	2	0	2	1	0	1	1	0	1	1	1	2	0	0	0	0	1	1	1
3	2	2	4	2	1	3	2	0	2	2	3	5	1	0	1	0	2	2	1
4	1	1	2	1	1	2	2	1	3	0	2	2	1	0	1	0	2	1	1
5	1	1	2	1	1	2	1	2	3	1	3	4	0	0	0	1	2	4	1
6	2	2	4	2	1	3	2	1	3	1	1	2	0	0	0	0	0	0	1
7	2	2	4	2	2	4	1	1	2	0	2	2	1	0	1	0	2	2	1
8	3	2	5	1	1	2	2	2	4	1	3	5	0	0	0	1	2	3	1
9	2	1	3	1	1	2	2	2	4	1	3	4	1	0	1	0	2	2	1
10	2	2	4	2	1	3	2	1	3	1	3	4	0	0	0	0	2	2	1
11	2	1	3	1	0	1	2	1	3	0	2	2	1	0	1	0	2	2	0
12	1	3	4	1	1	2	1	2	3	2	3	5	0	0	0	1	2	3	3
13	1	1	1	1	1	2	2	2	4	0	2	2	0	0	0	0	2	2	3
14	3	2	5	2	2	4	2	1	2	1	2	3	0	0	0	1	2	3	1
15	1	1	2	1	0	1	1	0	1	1	3	4	0	0	0	0	2	1	3
16	3	1	4	2	2	4	3	1	4	1	2	3	0	0	0	0	2	1	1

Legenda: LP: lobular-parenchimal; PP: portal-peribiliary; G: general; iNOS: inducible nitric oxide synthase; αSMA: alpha smooth muscle actin; Iba-1: ionized calcium binding adaptor molecule 1; NF-κB: Nuclear factor kappa-light-chain-enhancer of activated B cells.

**Table 6:** Individual IHC combined inflammation and fibrosis grades, considering hepatic areas.

N.	SUM IHC Inflammation LP (0–9)	SUM IHC Inflammation PP (0–9)	SUM IHC Inflammation G (0–9)	SUM IHC Fibrosis LP (0–9)	SUM IHC Fibrosis PP (0–9)	SUM IHC Fibrosis G (0–9)
1	6	0	6	5	4	9
2	4	0	4	1	2	3
3	6	3	9	3	5	8
4	4	3	7	1	4	5
5	3	4	7	2	5	7
6	6	4	10	1	1	2
7	5	5	10	1	4	5
8	6	5	11	2	5	7
9	5	4	9	2	5	7
10	6	4	10	1	5	6
11	5	2	7	1	4	5
12	3	6	9	3	5	8
13	4	4	8	0	4	4
14	7	5	12	2	4	6
15	3	1	4	1	5	6
16	8	4	12	1	4	5

Legenda: LP: lobular-parenchimal; PP: portal-peribiliary; IHC: immunohistochemistry; SUM IHC inflammation LP = Iba-1 LP grade + iNOS LP grade + NF-κB LP grade; SUM IHC inflammation PP = Iba-1 PP grade + iNOS PP grade + NF-κB PP grade; SUM IHC inflammation G = Iba-1 G grade + iNOS G grade + NF-κB G grade; SUM IHC fibrosis LP = αSMA LP grade + CD206 LP grade + Sirius Red LP grade; SUM IHC fibrosis PP = αSMA PP grade + CD206 PP grade + Sirius Red PP grade; SUM IHC fibrosis G = αSMA G grade + CD206 G grade + Sirius Red G grade.

## Data Availability

The complete dataset is available upon reasonable request.

## Abbreviations

The following abbreviations are used in this manuscript:

ALP	Alkaline phosphatase
ALT	alanine aminotransferase
AST	aspartate aminotransferase
BCS	Body Condition Score
BTD	Biliary Tract Disease
CD206	Macrophage Mannose Receptor
CLD	Chronic Liver Disease
FFPE	Formalin-Fixed Paraffin-Embedded
G	General
GGT	gamma-glutamyl transferase
HE	Hematoxylin-eosin
HSC	Hepatic Stellate Cell
Iba-1	Ionized Calcium Binding Adaptor Molecule 1
IHC	Immunohystochemistry
iNOS	Inducible Nitric Oxide Synthase
LP	Lobular-parenchymal
LPS	lipopolysaccharides
MASH	Metabolic Dysfunction Associated Steatohepatitis
MASLD	Metabolic Dysfunction Associated Steatotic Liver Disease
NAFLD	Non-alcoholic Fatty Lived Disease
NASH	Non-alcoholic Steatohepatitis
NF-κB	Nuclear factor kappa-light-chain-enhancer of activated B cells
PP	Portal-peribiliary
α-SMA	Alpha Smooth Muscle Actin
